# Supplementary material for: Impact of deforestation and temporal land-use change on soil organic carbon storage, quality, and lability
Source: PLoS One. 2022 Aug 10;17(8):e0263205. doi: 10.1371/journal.pone.0263205 (PMC9365162; doi:10.1371/journal.pone.0263205)
Supplement: Attachment — Submitted filename: Response to Reviewers Comments.docx [file pone.0263205.s002.docx]

**Response to Editor’s comments**

**Editor’s Comments:**

**Comment 1**:

Please consider changing the figure 1 adding the variable comparisons within measurements and not, as presently shown, the measurements close each other within each land use level. The reader needs to pay attention to the comparison per variable among treatment, not per treatment.

**Answer:**

Thanks. The purpose of showing figure 1 is to just show the chronology of events relative to the conversion of the native (primary) forest to the other land use types. Moreover, the drone-captured picture associated with the Figure 1 shows a pictorial overview of the existing land use types in the study area. Adding the variables considered in this study to figure 1 would make it clumsy and potentially defeat the purpose with which the figure is shown in the manuscript. Moreover, the existing land use picture in Figure 1 was captured by our DJI Mini 2 drone, which belongs to our program at The Ohio State University. So, there is no questions about downloading the picture from the website.

**Comment 2:**

Consider, if you agree, also using a multivariate analysis on the standardised variables you measured. Since you have replicates, I suggest the use of a canonical discriminant analysis (please mind to standardise variables before running it). Since you use SAS, please note that the SAS procedure for CDA is Proc Candisc.

**Answer:**

Thanks. As per your suggestion, we run the CDA (SAS and Orion), using all our replicated data including land use and soil depth; however, the results and graphical display were not understandable and visualized to our knowledge (CDA graph attached here).

Alternatively, we have used principal components (PCA) and redundancy analyses (RDA), which show satisfactory results and proper visualization of the relationships among the variables (added within the text of the manuscript. We have followed figures and graphs in few of your papers. I hope the PCA, and RDA will address the issues.

**Response to Reviewers**

**REVIEWER #1**

General
I read with interest the manuscript entitled “Impact of deforestation and temporal land-use change on soil organic carbon dynamic in a temperate ecosystem”. I think it falls in the aim and scopes of the journal.
From my point of view the title did not reflect the specific application, being very broad, it is not addressing the reader to the real topic of the manuscript which is an experimental trial with natural (forest)+specific perennial field crop, conventional tillage (please specify in the abstract).

Specific comments:

| **Comment/Suggestion** | **Response** |
| --- | --- |
| E 4: E 6 add ratio | Per suggestion, the revisions were made thoroughly within the text of the manuscript. Please see lines42, 50, 51, 64, 585, 586, 588, 663, 690, 693, 695, 696, 756. |
| The abstract is confusing, too much acronyms, no clear picture can be drawn. Please spend some time to tighten up the message. | The abstract was edited and concise. Few of the acronyms were taken out and all of them have been defined. A table (Table 1) was included in the M&M section to explain all the acronyms. |
| The study area is quite peculiar, I would like to see clearly the site effect and what will change if the catchment-area will be different. | The study area consists of all the sites on a relatively flat and homogeneous land except the differences in management systems over time. That is what we tried to show with regards to how management systems over time changed the site quality in terms of changes in soil organic carbon contents, quality, and lability, including other soil properties.  If the catchment area were different from the area where this study was done, there could be differences with regards to the site effect on the measured variables. Depending on the soil properties of the catchment area, land use type following the conversion of the primary forest, the level or amount of input used coupled with the magnitude of disturbance of the soil ecosystem, the carbon and nitrogen stoichiometry could be affected. |
| Language has some minor flaws (that could be addressed with proof reading), while few typos are due to inaccuracies in preparing the submission, most of them avoidable. | The manuscript has gone through a proof reading as much as we could do, and typo errors and grammatical inaccuracies have been corrected accordingly. |
| **Introduction**: Too broad in general, The introduction could be improved by looking at a scientific database like SCOPUS and perform a simple search to collect all studies about specific species used in the trial and similar environments | Thank you for your comment. The scope to this study was to understand the SOC pools and their lability, and how the SOC pools are impacted by land-use change to provide useful information on the land management implications for enhanced soil ecosystem functions and services. In as much as we appreciate the importance of collecting all studies on plant species studied under various ecosystems in similar temperate environments, we respectfully believe that it would not fit the scope of this study.  As suggested, few relevant literatures were cited to focus on the studies conducted under similar climates. |
| Avoid groups of references [maximum 2] if 3 please take a moment to explain what was so important and relevant to describe the third and so forth… | In all, groups of references have been reduced to a maximum of two, where applicable. Only in few cases, three references were cited to link and justify the results as there were updated terms and calculations used in this manuscript. |
| Line 139 impact | Revised, as suggested. Please see line 137. |
| Line 144 the word stratification is ambiguous, what would you like to say? Stratification is not the contrary of depletion, perhaps sequestration? | The word “stratification” used in this context is not ambiguous. Stratification is contrary to overall sequestration or depletion in response to the variable management systems and inputs when compared to the baseline or control systems deeper soil depth. It is not necessarily synonymous to sequestration, but it is an indicator of sequestration and defines the soil ecosystem functions and services (Franzluebbers et al. 2002). |
| Here and there are many terminology inaccuracies that needs revision | All the terminologies have been well defined. |
| **Material and methods** Please link the place in the title | Thank you for the suggestion. The title of the manuscript is simplified, as suggested. |
| Line 289 In the concentration of...(please specify) | The sentence is unambiguously clear. We looked at the concentrations (%, mg/kg, g/kg), stocks (Mg/ha), and stratification (unitless ratio) of soil organic carbon and total nitrogen pools. “Significant differences in the concentration, stocks, and stratification of SOC and TN pools…” Please see lines 287 – 289. |
| Line 293-294 is the LSD the best procedure to assess differences? | There are other statistical procedures to assess statistical differences between means in experimental trials. In this study, the widely used “Least Significant Difference (LSD) Test” was considered appropriate to elucidate the interactive effects of the predictor variables on dependent variables and to assess the mean differences between land use types relative to the respective measured variables.  Moreover, principal components- and redundancy analyses (PCA and RDA) were performed to improve the statistical validity and transferability of the results. |
| **Results:** fair | Revisions were made to improve flow and quality of the presentation. |
| **Discussion:** Please try to stress the relevant point, especially what can be done to ameliorate soils | Thanks. We focused on the evaluation of how the land use affected soil carbon pools and TN content over time. The CRP and other conservation management practices (such as establishment of bioenergy plantations under no-till) can be utilized to ameliorate the degraded and/or disturbed ecosystems with especial reference to soil quality and ecosystem services. More information was added in the manuscript. Please see lines 450 – 455. |
| **Conclusion:** try to give a take home message first | A take home message “stop deforestation and follow conservation practices such as CRP to sequester C and mitigate climate change effects” has been incorporated into the conclusion section of the manuscript. Please see lines 750 – 751; 761 – 764. |
| Line 746, why “temporal” | The word “temporal” has been deleted from the sentence. Please see line 747. |

**REVIEWER #2**

The manuscript addresses a subject of current interest related to the effect of temporal land-use change, from forest to agriculture, wetland, CRP on Soil organic carbon, and total nitrogen Stocks in the temperate climate. Such information in temperate regions is scarce compared to the tropical regions. The novelty of this research, compared to other research when studying the spatial or temporal change of land on SOC and TN stock, is the advanced analysis of carbon and nitrogen pools, namely the humic substances (humic and fulvic acids, humin), the active carbon, the use of humification index (HI), humification ratio (HR), degree of humification (DH), carbon pool index (CPI), nitrogen pool index (NPI), and carbon management index (CMI) to better assess the impacts. Overall, I feel the study can be suitable for publication in PLOS ONE after minor revision.

Some suggestions for further improvement are given below:

| **Comment/Suggestion** | **Response +Page/Line numbers** |
| --- | --- |
| Page 10 Line 232: please define the parameters and their unit used to calculate C or N Stocks. | Thanks. Revisions were performed, as suggested through-out the manuscript. Please see lines 206 – 207. |
| Page 18 Line 464: ”Plowing promotes nutrient mineralization under warmer and oxic soil conditions”: Please correct the grammar mistake | The sentence has been revised. Please see Lines 445 – 448. |
| Page 22 Line 572: ”the TN and humin stratification patterns we observed were similar to that of SOC.” Please reformulate the sentence. | The sentence is reformulated to make it comprehensible. Please see Line 554. |
| The quality of the Figures 1, 2, 3, 4, 5, and 6 is too low, it is not possible to read them, please improve the quality of these figures. | Thank you for your suggestion. The quality of the figures has been improved upon. |
